# LncRNAs regulate cancer metastasis via binding to functional proteins

**DOI:** 10.18632/oncotarget.22840

**Published:** 2017-12-01

**Authors:** Liting Yang, Yanyan Tang, Fang Xiong, Yi He, Fang Wei, Shanshan Zhang, Can Guo, Bo Xiang, Ming Zhou, Ni Xie, Xiaoling Li, Yong Li, Guiyuan Li, Wei Xiong, Zhaoyang Zeng

**Affiliations:** ^1^ The Key Laboratory of Carcinogenesis of the Chinese Ministry of Health, Xiangya Hospital, Central South University, Changsha, Hunan, China; ^2^ The Key Laboratory of Carcinogenesis and Cancer Invasion of the Chinese Ministry of Education, Cancer Research Institute, Central South University, Changsha, Hunan, China; ^3^ Hunan Key Laboratory of Nonresolving Inflammation and Cancer, Disease Genome Research Center, The Third Xiangya Hospital, Central South University, Changsha, Hunan, China; ^4^ Hunan Key Laboratory of Translational Radiation Oncology, Hunan Cancer Hospital and The Affiliated Cancer Hospital of Xiangya School of Medicine, Central South University, Changsha, Hunan, China; ^5^ Core Laboratory, Shenzhen Second People’s Hospital, First Affiliated Hospital of Shenzhen University, Shenzhen, Guangdong, China; ^6^ Department of Cancer Biology, Lerner Research Institute, Cleveland Clinic, Cleveland, Ohio, USA

**Keywords:** lncRNA, functional proteins, metastasis, localization, stabilization

## Abstract

Cancer is one of the leading causes of death worldwide, and metastasis is a crucial characteristic of malignancy. Recent studies have shown that lncRNAs play an important role in regulating cancer metastasis through various molecular mechanisms. We briefly summarize four known molecular functions of lncRNAs, including their role as a signal, decoy, guide and scaffold. No matter which pattern lncRNAs follow to carry out their functions, the proteins that lncRNAs bind to are important for them to exhibit their gene-regulating properties. We further illustrate that lncRNAs regulate the localization, stabilization or modification of their binding proteins to realize the binding role of lncRNAs. In this review, we focus on the interactions between lncRNAs and their binding proteins; moreover, we focus on the mechanisms of the collaborative work of lncRNAs and their binding proteins in cancer metastasis, thus evaluating the potential of lncRNAs as prospective novel therapeutic targets in cancer.

## BACKGROUND

Cancer is one of the leading causes of death worldwide, and as one of the hallmarks of cancer, metastasis is a crucial characteristic of malignancy [1, 2]. Metastasis is a complex multistep process that involves the early invasion and late colonization of cancer cells [3]. Usually, cancer cells undergo morphological alterations and change their cell-cell or cell-matrix interactions to be able to successfully pass through the first steps of the multistep process of metastasis [4–9]. EMT (epithelial-mesenchymal transition) is of critical importance in the early events of tumor cell metastatic dissemination by endowing the cells with a more motile, invasive potential [10–15]. On the other hand, MET (mesenchymal-epithelial transition) is required for migrating cells to extravasate from the vessels into their target tissues to form micrometastases and eventually form a secondary tumor after the cells survive anoikis [16].

LncRNAs (long non-coding RNAs) are a class of transcripts longer than 200 nucleotides with limited protein coding potential [17–22]. In the past, extensive efforts have been made to characterize the involvement of protein-coding genes in cancer metastasis but few for lncRNAs, which have once been thought as the “dark matter” of the genome, because of our limited knowledge about their functions. Up to now, more and more studies have identified that deregulations of lncRNAs are observed in various cancer types, and an abnormal expression of lncRNAs virtually participates in all stages of cancer development, including cancer initiation, progression and metastasis [23–29].

Various modes of molecular interactions were observed for lncRNAs to exhibit their gene-regulating properties in the recent years. Studies indicate that lncRNAs bind to proteins and regulate their functions. In this review, we summarize the role of lncRNAs binding to functional proteins to regulate their localization, stabilization or modification in cancer metastasis.

### LncRNAs regulate cancer metastasis through multiple signaling pathways

**Figure 1:**
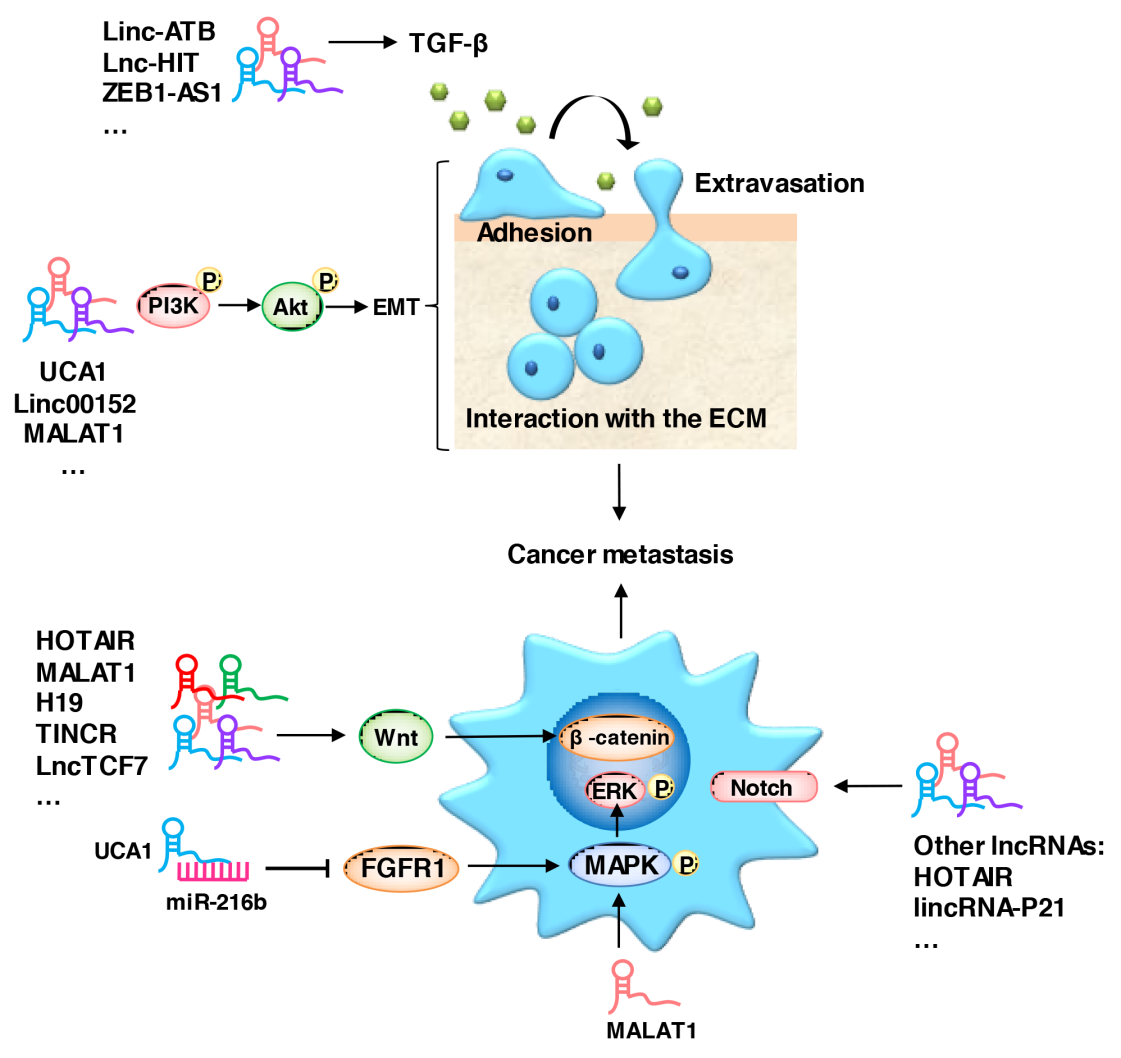
LncRNAs act as regulators of metastasis-related signaling pathways An aberrant expression of lncRNAs promotes cancer metastasis via the TGF-β/SMAD, WNT/β-catenin, NOTCH, MAPK/ERK, PI3K/Akt and HEDGEHOG signaling pathways.

Metastasis of cancer cells to a distal site is a particularly critical stage of cancer progression [30–36]. EMT is a critical process during the initiation of cancer metastasis, the changes in this program includes cell morphology, cell-matrix adhesion and migration abilities. E-cadherin, intergrins, and cytokeratins are the most commonly used epithelial markers, while N-cadherin, vimentin and fibronectin are mesenchymal markers [10]. A group of EMT-inducing transcription factors, including Slug, Snail and ZEB1/2 (zinc finger E-box binding homeobox 1/2), are activated during EMT [37]. Growth signals from the tumor stroma, such as TGF-β (transforming growth factor) [38–41], EGF (epidermal growth factor), FGF (fibroblast growth factor), PDGF (platelet-derived growth factor), IGF (insulin growth factor) and HGF (hepatocyte growth factor), are responsible for triggering EMT in cancer cells [42–45]. These inducers trigger EMT through a complex signaling network, including several receptor tyrosine kinases (RTKs) and the TGF-β/SMAD, WNT/β-catenin, NOTCH, MAPK/ERK, PI3K/Akt and HEDGEHOG signaling pathways [46–60]. The aberrant expression of lncRNAs plays a considerable role in cancer metastasis, and they have emerged as versatile regulators of the EMT related pathways mentioned above (Figure 1).

The TGF-β signaling pathway is one of the major pathways responsible for the induction of EMT via a group of transcription factors, including Slug, Snail and Twist [61, 62]. *LncRNA-HIT* (LncRNA-HOXA transcript induced by TGF-β) is one of the most upregulated lncRNAs induced by TGF-β [63]. The upregulation of *LncRNA-HIT* promotes the migration and invasion of NSCLC by directly associating with ZEB1 [64]. The knock down of *lncRNA-ATB* impedes the induction of EMT by TGF-β in HCC and colon cancer [65, 66]. *ZEB2-AS1*, *MALAT1* and *linc01133* also play a critical role in the TGF-β signaling pathway [67–69].

The canonical WNT/β-catenin pathway also plays an important role in the regulation of cancer metastasis [70, 71]. β-catenin is a core component of the cadherin protein complex, whose localization and stabilization are essential for the activation of WNT/β-catenin signaling [72, 73]. Several lncRNAs are involved in WNT/β-catenin pathway regulation. For example, *HOTAIR* (HOX transcript antisense RNA) epigenetically silences the Wnt inhibitor WIF1, while the loss of WIF1 enhances the migratory ability of glioblastoma cells through WNT5A activation mediated via *MALAT1* [74, 75]. The *lncRNA H19*, *TINCR*, and *lncTCF7* also play critical roles in the regulation of cancer metastasis via the WNT/β-catenin pathway [76–78].

MAPK/ERK signaling is an element in lncRNAs that mediates the regulation of metastasis. *MALAT1* promotes the metastasis of gallbladder carcinoma through the activation of the MAPK/ERK pathway [79]. *UCA1* (lncRNA urothelial carcinoma-associated 1) plays a pivotal role in the tumorigenesis of HCC by acting as a ceRNA for miR-216b, leading to the suppression of FGFR1 (fibroblast growth factor receptor 1) expression and the activation of the MAPK/ERK signaling pathway [80].

In addition to their roles in the pathways mentioned above, several lncRNAs also utilize the PI3K/Akt signaling pathway in metastasis regulation. *Linc00152* directly binds with EGFR, which activates PI3K/Akt signaling in gastric cancer [81]. The downregulation of *MALAT1* induces EMT via the PI3K/Akt pathway in breast cancer [82], and in contrast, the upregulation of MALAT1 promotes the metastasis of osteosarcoma cells by activating the PI3K/Akt pathway [83].

LncRNAs regulate metastasis using additional pathways, such as the Notch and Hedgehog signaling pathways [84–88]. There are so many patterns for lncRNAs to regulate cancer metastasis, and thus, a particular focus on the molecular mechanisms of lncRNAs in metastasis is needed.

### Roles of lncRNAs in regulating protein functions

LncRNAs use various modes of molecular interactions to exhibit their gene-regulating properties from transcriptional to posttranscriptional regulation. The functional domains of lncRNAs include RNA-binding domains, DNA-binding domains and protein-binding domains [89]. Thanks to structural plasticity, lncRNAs can act as signals, decoys, guides and scaffolds [90].

LncRNAs show a type-specific expression and respond to various stimuli, suggesting that lncRNAs can serve as molecular signals [90]. As signals, lncRNAs mark space, time, and expression for gene regulation. For instance, *lincRNA-p21* is a transcriptional target of p53 and plays a role in triggering apoptosis [91]. Other lncRNAs, such as *HOTAIR* [92] and *Xist* (X-inactive-specific-transcript) [93], also function as signals, and they usually serve as markers of biological events and are capable of delivering further signals.

LncRNAs act as molecular decoys, because they possess an RNA motif, which binds and titrates away proteins or RNA targets. Some lncRNAs, acting as a “sponge” of miRNAs, also belong to this archetype. In this type, lncRNAs function as CeRNAs (competing endogenous RNA), and vie with mRNAs for miRNAs with shared MREs (miRNAs responses elements) and act as a modulator of miRNAs by influencing the available level of miRNAs [94, 95]. Sponge LncRNAs, such as *H19* [64, 96–99], *HOTAIR* [100–102], MALAT1 [69, 103–107], *UCA1* [108–111], *XIST* [112, 113], *HULC* [114], *lincRNA-ROR* [115], *lnc-FTX* [116], *NEAT1* [117, 118], *SNHG1* [119], *TUG1* [120], and *TUSC7* [121], are typical examples.

The third molecular role of lncRNAs is a guide. By acting as a guide, lncRNAs bind to proteins and then direct the localization of the resultant complex to specific targets [90]. Guide lncRNAs directly interact with DNA and RNA by base pairing, and highly structured lncRNAs also provide docking sites for binding proteins [122]. For example, lncRNA *HOTTIP* directly binds the adaptor protein WDR5 and targets WDR5/MLL complexes to the *HOXA*, increasing the H3 lysine 4 trimethylation of the HOXA cluster to cause gene transcription [90]. There are many other lncRNAs that function as guides, such as *HOTAIR* [92] and *SChLAP1* [123].

The fourth function of lncRNAs is a scaffold. LncRNAs serve as central platforms on which different effector molecules are assembled. The function of LncRNAs as scaffolds is perhaps the most functionally intricate and complex class, in which the lncRNAs possess different domains that bind distinct effector molecules [90]. One example is Kcnq1ot1, which binds both PRC2 and G9a to promote H3K27me3 and H3K9me3 [124]. Other lncRNAs such as *ANRIL* (CDKN2B antisense RNA 1) [125, 126], *HOTAIR* [127], and *GClnc1* (gastric cancer-associated lncRNA 1) [128] also function as scaffolds to regulate gene expression.

As a whole, the archetypes mentioned above are not mutually exclusive. LncRNAs play regulatory functions through either RNA-protein or RNA-DNA recognition rules. No matter which archetype lncRNAs carry out their functions, the proteins they bind to are important for them to exhibit their metastasis-regulating performances. In general, lncRNAs complex with various proteins to regulate their localization, stabilization or modification.

### LncRNAs regulate the localization of binding proteins

**Figure 2:**
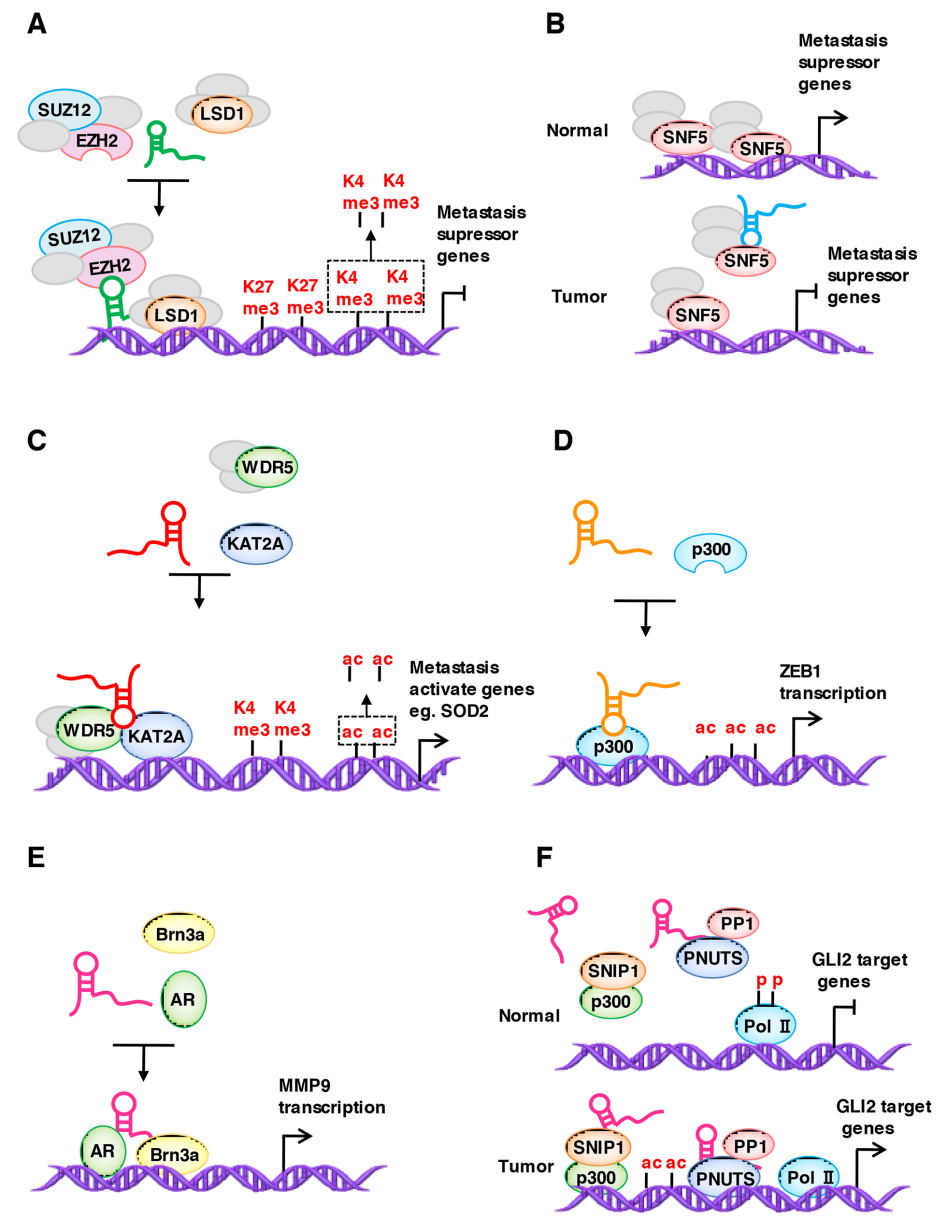
LncRNAs regulate the localization of the chromatin modification complex **(A)** HOTAIR functions as a molecular scaffold to link and target PRC2 and LSD1, which then reprograms chromatin states by coupling histone H3K27 methylation and H3K4 demethylation for epigenetic gene silencing to promote cancer metastasis. **(B)** SChLAP1 interacts with SNF5 and functions as a molecular decoy that sequesters the SWI/SNF chromatin-modifying complex away from selective gene loci to regulate gene expression. **(C)** GClnc1 upregulates the transcription of SOD2 by acting as a scaffold to recruit the WDR5 and KAT2A complex to the SOD2 promoter, increasing H3K4 trimethylation and H3K9 acetylation levels in the SOD2 promoter region. **(D)** ZEB1-AS1 directly binds and recruits p300 to the ZEB1 promoter, which induces an open chromatin structure and activates ZEB1 transcription. **(E)** SLNCR1 binds to AR and Brn3a. The SLNCR1/AR/Brn3a ternary complex, located upstream of the MMP9 transcription start site, increases MMP9 expression. **(F)** BCAR4 binds to SNIP1 and PNUTS. In response to cytokine stimulation, BCAR4 lifts the inhibitory effect of SNIP1 on p300, leading to the acetylation of histones, which in turn leads to the activation of polymerase II at GLI2 controlled genes.

Chromatin-modifying proteins are a major group of lncRNA binding proteins, and lncRNAs bind to and guide them to specific sites in the genome to regulate metastasis-related genes expression in space and time. A prominent example of a histone-modifying complex interacting with lncRNAs is PRC2, a histone methyltransferase that catalyzes the mono-, di- and trimethylation of H3K27, which is required for epigenetic silencing during development and cancer. The core PRC2 complex is composed of four proteins, including EZH1/2, SUZ12, EED and RbAP46/48 [129]. Until now, the question of how the histone-modifying complex identifies its binding sites on the chromatin remains open, and studies on lncRNAs may give us new and exciting answers. *HOTAIR*, a lncRNA first identified by Rinn et al., interacts with PRC2 and is required for PRC2 occupancy on the HOXD locus [92]. Later, researchers found that *HOTAIR* is upregulated in different kinds of cancers and promotes cancer metastasis through by regulating the localization of PRC2 [130–133]. Mechanistically, HOTAIR recruits the PRC2 complex to specific target genes genome-wide, leading to H3K27 trimethylation and epigenetic silencing of metastasis suppressor genes. LncRNAs also functions as molecular scaffold to link PRC2 and other modification proteins [127, 134–136]. For example, HOTAIR functions as a molecular scaffold to link and target the histone modification complexes PRC2 and LSD1 and then reprograms chromatin states by coupling histone H3K27 methylation and H3K4 demethylation for epigenetic gene silencing to promote cancer metastasis (Figure 2A).

Another example is *SChLAP1*, which is a lncRNA upregulated in prostate cancer. *SChLAP1* promotes prostate cancer invasiveness and metastasis by binding to SWI/SNF and titrating it away from the chromatin [123]. The mammalian SWI/SNF complex mediates ATP-dependent chromatin remodeling processes, and a substantial of evidence indicates that several components of the SWI/SNF complexes function as tumor suppressors [137]. In detail, *SChLAP1* interacts with SNF5 (also known as SMARCB1, an essential subunit that facilitates SWI/SNF binding to histone proteins) and functions as a molecular decoy that sequesters the SWI/SNF chromatin-modifying complex away from the selective gene loci to inhibit metastasis suppressor gene expression (Figure 2B).

MLL1 is a member of the evolutionarily conserved SET1 family of histone H3 lysine4 (H3K4) methyltransferases, which are required for the regulation of distinct groups of developmentally regulated genes [138, 139]. WDR5 is a core subunit of MLL1 and acts as an “effector” of H3K4 methylation in gene transactivation [128]. Sun et al. identified a novel lncRNA, *GClnc1*, which promotes gastric cancer metastasis [106]. In detail, *GClnc1* upregulates the transcription of *SOD2* (dismutase 2 mitochondrial) by acting as a scaffold to recruit the WDR5 and KAT2A (histone acetyltransferase) complex to the *SOD2* promoter and increasing the H3K4 trimethylation and H3K9 acetylation levels in the *SOD2* promoter region (Figure 2C). Upregulated *SOD2* expression consequently promotes metastasis.

p300 is a HAT (histone acetyltransferase) member that acetylates histone proteins by transferring an acetyl group from acetyl-CoA to specific lysine residues [140]. The acetylation of histones by HATs results in a dispersed structure of chromatin, which becomes accessible to transcriptional factors [141]. *ZEB1-AS1* promotes cell migration in osteosarcoma by directly binding and recruiting p300 to the *ZEB1* promoter, which induces an open chromatin structure and activates *ZEB1* transcription [142] (Figure 2D).

In addition to chromatin-modifying proteins, transcription factors also interact with lncRNAs. A study identified that lncRNAs guide transcription factors to specific sites in the genome. For example, *SLNCR* (SRA-like non-coding RNA) contains a conserved∼300 nucleotide region with a significant similarity to steroid receptor RNA activator 1 (SRA1). Schmidt et al. [143] reported that Brn3a (a member of the Brn 3 family of POU-domain transcription factors) and AR (a steroid-hormone activated transcription factor) bind to *SLNCR1*’s conserved sequence and an adjacent sequence, respectively. The SLNCR1/AR/Brn3a ternary complex has a high affinity for the AR and Brn3a binding sites located upstream of the *MMP9* transcription start site, and the cooperative binding of AR and Brn3a to its promoter increases *MMP9* expression and activity and, thus, increases the invasion of melanoma cells (Figure 2E). Another prominent example is *BCAR4* (breast cancer anti-estrogen resistance 4), which contributes to tumor metastasis by binding to SNIP1 (SMAD nuclear interacting protein 1) and PNUTS (a phosphatase). In response to cytokine stimulation, *BCAR4* lifts the inhibitory effect of SNIP1 on p300, leading to the acetylation of histones, such as H3K18ac. Acetylated histones are necessary for the BCAR4-mediated recruitment of PNUTS, which in turn leads to the active polymerase II at GLI2 controlled genes [144] (Figure 2F).

The lncRNAs mentioned above guide the binding proteins to chromatin or titrate the binding proteins away from the chromatin to regulate gene expression directly. LncRNAs can also decoy the protein from it RNA targets or interact proteins. For example, *LINC01133*, which is downregulated by TGF-β, inhibits EMT and metastasis in colorectal cancer [69]. Mechanistically, *LINC01133* acts as key downstream molecule in the TGF-β pathway and inhibits the EMT in colorectal cancer by directly binding to SRSF6 (serine and arginine rich splicing factor 6) as a target mimic. The authors speculate that *LINC01133* may act as a decoy element, titrating SRSF6 away from it RNA targets by directly binding to its critical domain to block the induction of EMT. However, how SRSF6 regulates colorectal cancer cell EMT is still unknown. Another study found that *MALAT1* promotes colorectal cancer cell metastasis by competitively binding to PSF and releasing SFPQ from the SFPQ/PTBP2 (polypyrimidine tract binding protein 2) complex, which then increases the SFPQ-detached proto-oncogene PTBP2 [145].

### LncRNAs regulate the stabilization of binding proteins

Like all macromolecular components of an organism, the proteome is in a dynamic state of synthesis and degradation. The proteolytic equilibrium of the proteins is disturbed and the microenvironment is changed in pathophysiological conditions [146]. Studies find that lncRNAs play an important role in regulating the stabilization of binding proteins.

**Figure 3:**
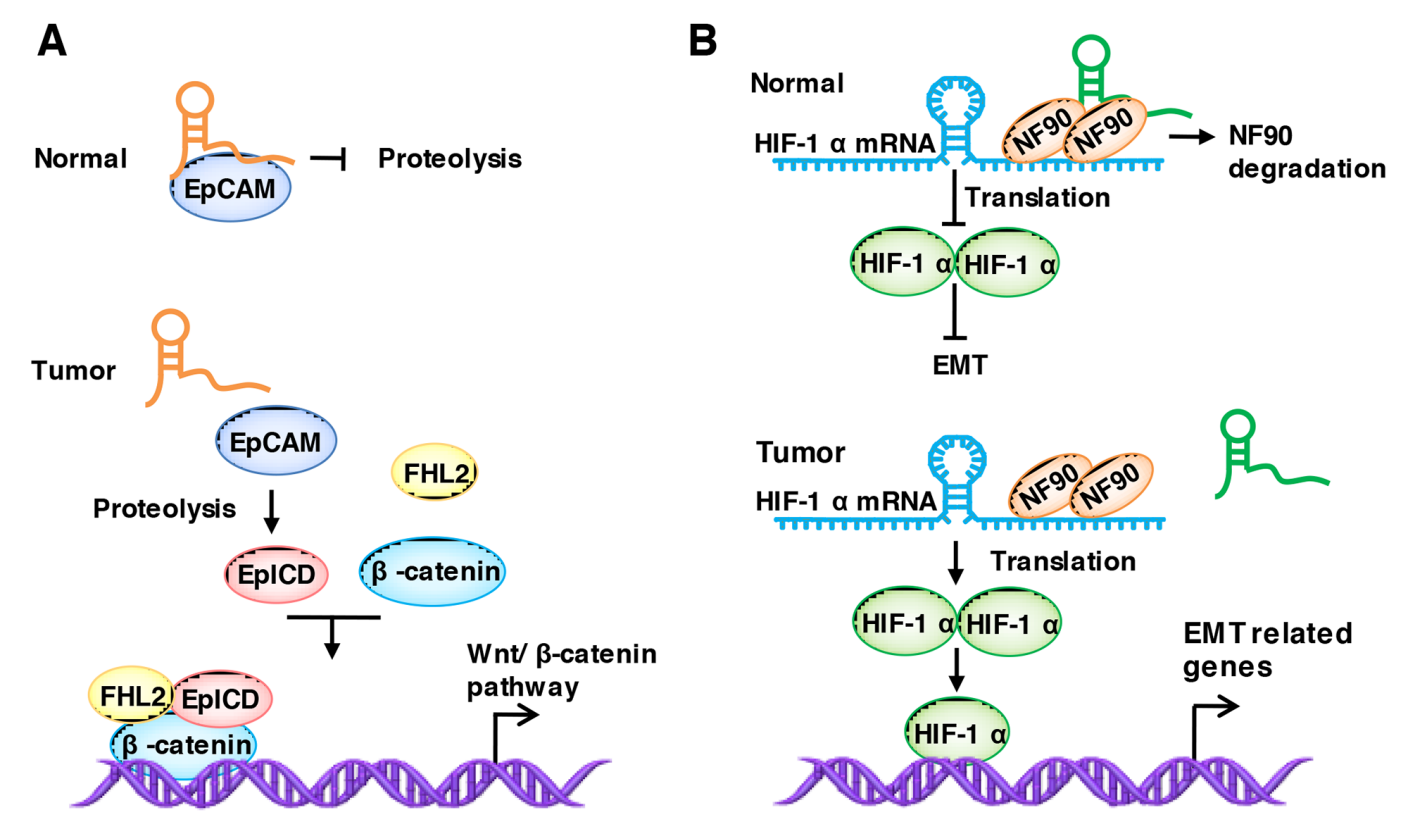
LncRNAs regulate the stabilization of binding proteins **(A)** TINCR binds to EpCAM and prevent its proteolysis. The loss of TINCR promotes the hydrolysis of EpCAM and releases EpICD. EpICD colocalizes with FHL2 and β-catenin to form a nuclear protein complex, leading to gene transcription. **(B)** LncRNA-LET binds to NF90 and enhances its degradation, thereby affecting HIF-1α mRNA accumulation and stability under hypoxic conditions, and the inactivation of HIF-1α results in the decreased expression of EMT-related proteins, thus leading to the inhibition of EMT, motility and invasiveness.

*TINCR* (Terminal differentiation-induced lncRNA) was first reported by Markus et al. [147], and they declared that the 3.7-kilobase lncRNA controls human epidermal differentiation by a post-transcriptional mechanism. Then, Zhang et al. [77] found that the loss of *TINCR* expression promotes colorectal cancer metastasis by specifically binds to EpCAM, preventing its proteolysis. EpCAM is expressed at the basolateral membrane of most normal epithelial cells but is over-expressed in many epithelial cancers. The loss of *TINCR* promotes the hydrolysis of EpCAM and then releases EpICD (EpCAM c-term, intracellular domain), which is one of the components of the Wnt pathway, and colocalizes with FHL2 and β-catenin to form a nuclear protein complex, leading to gene transcription and, subsequently, activating the Wnt/β-catenin pathway [77, 148] (Figure 3A).

*LncRNA-LET* inhibits the metastasis of HCC and colorectal cancer cells, which are suppressed by HDAC3 (histone deacetylase 3) in hypoxia conditions [149]. A study found that *lncRNA-LET* functions through its association with NF90, which is a double-strand RNA-binding protein that has been implicated in the stabilization, transport, and translational control of many target mRNAs, including *HIF-1α* (hypoxia-inducible factor 1 alpha subunit) [150]. Mechanistically, *lncRNA-LET* binds to NF90 and enhances its degradation, which thereby affects *HIF-1α* mRNA accumulation and stability under hypoxic conditions, and the inactivation of *HIF-1α* results in the decreased expression of EMT-related proteins, leading to an inhibition of EMT, motility and invasiveness [121, 149] (Figure 3B).

*LncRNA-HIT* promotes metastasis in NSCLC (non-small cell lung cancer) via specially binding to ZEB1 [64]. ZEB factors contain multiple domains that interact with other transcription factors, which is essential for the regulation of EMT [151, 152]. Mechanistically, the association between *LncRNA-HIT* and ZEB1 protects ZEB1 from proteasome degradation. Upregulated *LncRNA-HIT* promotes migration and invasion via increasing the occupancy of ZEB on the promoter region of *CDH1* [64]. LncRNA *AOC4P* (amine oxidase, copper containing 4, pseudogene) is another lncRNA that regulates the EMT marker protein directly. LncRNA *AOC4P* suppress EMT in HCC by binding to vimentin, the major component of the cytoskeleton, and enhancing its degradation [153].

### LncRNAs regulate the post-translational modification of binding proteins

**Figure 4:**
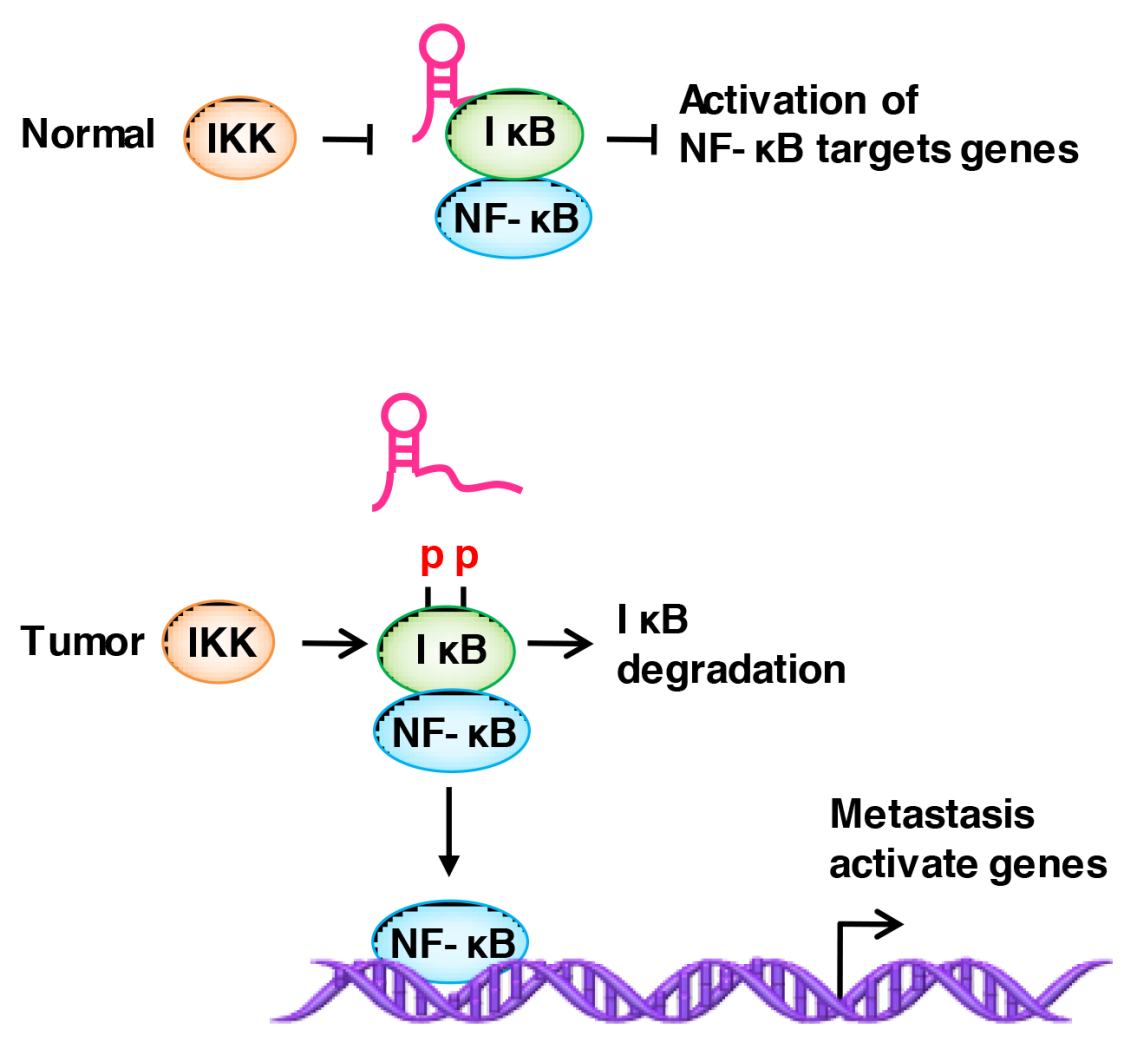
LncRNAs regulate the modification of binding proteins NKILA binds to the NF-kB / IkB complex and directly masks the phosphorylation motifs of IkB, thereby inhibiting IKK-induced IkB phosphorylation and NF-kB activation.

NF-kB is a family of transcription factors, and aberrant NF-kB activation promotes cancer invasion and metastasis [154–156]. Liu et al. [157] identified the lncRNA *NKILA* (NF-KappaB Interacting LncRNA), which has a low expression in breast cancer and binds to the NF-kB / IkB complex. IkB (inhibitor of NF-kB) acts as a negative regulator of NF-kB by binding to and sequestering NF-kB in the cytoplasm. It is considered as a major brake in NF-kB signaling [154]. *NKILA* binds to the NF-kB / IkB complex and directly masks the phosphorylation motifs of IkB, which thereby inhibits IKK-induced IkB phosphorylation and NF-kB activation [133] (Figure 4).

In addition to the three patterns lncRNAs use to regulate their binding proteins, lncRNA regulate binding proteins through other way. A high expression of GAPLINC (Gastric adenocarcinoma predictive long intergenic noncoding RNA) promotes colorectal cancer invasion by binding to PSF (also known as SFPQ, splicing factor proline and glutamine rich) and NONO (non-POU-domain-containing, octamer binding) [158]. A further study found that PSF and NONO promote GAPLINC to influence cell invasion partly by increasing the expression of SNAI2 (snail family zinc finger 2), a member of the snail family of transcription factors, which promotes cell invasion, motility, and metastasis via inhibiting E-cadherin transcription and inducing EMT in several human cancers [158–161]. However, how PSF and NONO bound with GAPLINC and how they activate GAPLINC-associated genes to promote invasion is still unknown.

## CONCLUSION

Currently, studies on lncRNAs have gradually become one of the hottest topics in the field of RNA biology, and lncRNAs have emerged as a versatile regulator of key pathophysiological pathways. LncRNAs have broad applications in cancer diagnosis and treatment because most of the well-studied lncRNAs are correlated with a poor prognosis in patients. The abnormal expression of lncRNAs in cancers reminds us they may be the targets of tumor diagnosis and treatment. Studies also show that lncRNAs take part in regulating cancer chemotherapy sensitivity. For example, *HOTAIR* activates the PI3K/Akt pathway by inhibiting the expression of miR-126 and promotes the development of cisplatin resistance in gastric cancer [162], while *HOTAIR* decreases chemoresistance through the activation of Wnt/β-catenin signaling in ovarian cancer [80]. Other lncRNAs, such as *Linc00152* [163], *MALAT1* [164], *UCA1* [165, 166] and *lnc-ROR* [167], are associated with chemotherapy sensitivity.

In addition, lncRNAs detected in the blood may represent prominent novel biomarkers for cancer diagnostics. For example, *HOTAIR* is detected in colorectal cancer and represents an effective negative prognostic biomarker for colorectal cancer in blood samples [168]. *MALAT1* is elevated in the whole blood of metastatic lung cancer patients [169], and *HULC,* detected in the blood, is also proposed as a diagnostic biomarker both for liver cancer and gastric cancer [170, 171]. Studies that illuminate the molecular mechanism of the abnormal expression of lncRNAs in various cancers are helpful to improve the efficiency of clinical treatments and the diagnosis of cancer.

More and more lncRNAs, with a differential expression in tumors, are being discovered, and these lncRNAs are important regulators of genes during cell metastasis or act as regulators for other metastasis-relevant genes. Up to now, two major mechanisms have emerged for how lncRNAs regulate cancer metastasis, including (1) binding to functional proteins characteristically and then affecting the transcription of genes associated with metastasis and (2) acting as ceRNAs for miRNAs that target genes involved in metastasis regulation. There are three control modes for lncRNAs to bind to functional proteins, including (1) by regulating the localization of binding proteins, lncRNAs play a role in the chromatin and epigenetic modification and the transcription of metastasis-relevant genes, (2) lncRNAs enhance or attenuate protein stability by binding to them, and (3) lncRNAs mask or expose the modification motif to inactivate or activate the binding protein.

In summary, this review highlights the interactions between lncRNAs and their binding proteins and the mechanisms of their collaborative roles in cancer metastasis (Table 1), which provides systematic information and an evaluation of the potential of lncRNAs as prospective novel therapeutic targets in cancer.

**Table 1:** Summary of lncRNAs and their binding proteins as regulators of cancer metastasis

Symbol	Interaction protein	Archetype	Mechanism	Cancer type	References
HOTAIR	PRC2	Guide	Histone modification; Promote the transcription of ABL2, SNAIL et al.)	Breast cancer	[130]
	PRC2 (EZH2)	Guide	Histone modification; Repress E-cadherin transcription	OSCC	[133]
	PRC2 (SUZ12)	Guide	Histone modification; Suppress promoter methylation of PCDH10	GISTs	[132]
	PRC2	Guide	Histone modification; HOTAIR-miR34a→ HGF/C-Met/Snail pathway	Gastric cancer	[131]
	PRC2, LSD1	Scaffold	Histone modification	-	[127]
NBAT1	PRC2 (EZH2)	Guide	Histone modification; Promote the transcription of DKK1	Breast cancer	[172]
DANCR	PRC2 (EZH2)	Guide	Histone modification; Suppress the transcription of TIMP 2/3	Prostate cancer	[173]
LINC00511	PRC2 (EZH2)	Guide	Histone modification; Repress p57 expression	NSCLC	[134]
Linc-UBC1	PRC2 (EZH2, SUZ12)	Guide	Histone modification	Bladder cancer	[174]
LncRNA-EBIC	PRC2 (EZH2)	Guide	Histone modification; Repress E-cadherin transcription	Cervical cancer	[175]
HOXA11-AS	EZH2, LSD1 or DNMT1	Scaffold, decoy	Histone/DNA modification “Sponge” of miR-1297	Gastric cancer	[134]
LncRNA-GIHCG	EZH2, DNMT1	Scaffold	Histone/DNA modification; Silence the expression of miR200 b/a/429	HCC	[135]
AGAP2-AS1	EZH2, LSD1	Scaffold	Histone modification; Repress LATS2 and KLF2 transcription	NSCLC	[136]
SChLAP1	SWI/SNF (SNF5)	Decoy	Histone modification;	Prostate cancer	[123]
GClnc1	WDR5, KAT2A	Scaffold	Histone modification; Promotes SOD2 transcription	Gastric cancer	[106]
ZEB1-AS1	p300	Guide	Histone modification; Activates ZEB1 transcription	Osteosarcoma	[142]
SLNCR1	Bm3a, AR	Scaffold Guide	Activates MMP9 transcription	melanoma	[143]
BCAR4	SNIP1, PNUTS	Scaffold	CCL21→BCAR4→non-canonical Hedgehog/GLI2 pathway	Breast cancer	[144]
LINC01133	SRSF6	Decoy	-	Colorectal cancer	[69]
MALAT1	PSF	Decoy	Release oncogene PTBP2 from PSF/PTBP2 complex	Colorectal cancer	[145]
	PRC2 (EZH2)	Guide Decoy	Repress E-cadherin expression “Sponge” of miR-1297	Colorectal cancer	[176]
	PRC2 (EZH2)	Guide	Histone modification; Repress PCDH10 expression	Gastric cancer	[177]
	PRC2 (SUZ12)	Guide Signal	Histone modification; Repress E-cadherin expression	Bladder cancer	[68]
TINCR	EpCAM	Signal	TINCR→ hydrolysis of EpCAM → EpICD→Wnt/β-catenin pathway	Colorectal cancer	[77]
LncRNA-LET	NF90	Signal	LncRNA-LET→ NF90 degradation -HIF-1α	HCC	[149]
LncRNA-HIT	ZEB1	Signal	Protect ZEB1 from degradation and then Repress E-cadherin expression	NSCLC	[64]
AOC4P	Vimentin	Signal	Enhance vimentin degradation	HCC	[153]
ANCR	EZH2	Signal	Enhance the degradation of vimentin	Breast cancer	[178]
NKILA	NF-κB/IκB	Signal	Blocks IkB phosphorylation	Breast cancer	[133]
CCAT2	EZH2	Guide	Histone modification; Repress E-cadherin /LATS2 expression	Gastric cancer	[179]
Lnc TCF7	SWI/SNF	Guide	Histone modification; Activate Wnt/β-catenin pathway	HCC	[78]
HNF1A-AS1	DNMT1	Guide	DNA modification; Repress E-cadherin expression	Lung adenocarcinoma	[180]
HULC	EZH2	Guide	Histone modification; Repress NKD2 expression	Colorectal cancer	[181]

## Abbreviations

EMT: epithelial-mesenchymal transition
MET: mesenchymal-epithelial transition
lncRNAs: long non-coding RNAs
HOTAIR: HOX transcript antisense RNA
Xist: X-inactive-specific-transcript
CeRNAs: competing endogenous RNA
MREs: miRNAs responses elements
MALAT1: metastasis associated lung adenocarcinoma transcript 1
UCA1: urothelial cancer associated 1
HULC: hepatocellular carcinoma up-regulated long non-coding RNA
NEAT1: nuclear paraspeckle assembly transcript 1
SNHG1: small nucleolar RNA host gene 1
SCHLAP1: SWI/SNF complex antagonist associated with prostate cancer 1
ANRIL: CDKN2B antisense RNA 1
GClnc1: gastric cancer-associated lncRNA 1
SOD2: dismutase 2 mitochondrial)
HATs: histone acetyltransferases
SLNCR: SRA-like non-coding RNA
BCAR4: breast cancer anti-estrogen resistance 4
SNIP1: SMAD nuclear interacting protein 1
SRSF6: serine and arginine rich splicing factor 6
PTBP2: polypyrimidine tract binding protein 2
TINCR: Terminal differentiation-induced lncRNA
HDAC3: histone deacetylase 3
*HIF-1α*: hypoxia-inducible factor 1 alpha subunit
LncRNA-HIT: HOXA transcript induced by TGF-β
NSCLC: non-small cell lung cancer
ZEB1: zinc finger E-box binding homeobox 1
AOC4P: amine oxidase, copper containing 4, pseudogene
NKILA: NF-KappaB Interacting LncRNA
IkB: inhibitor of NF-kB
GAPLINC: Gastric adenocarcinoma predictive long inergenic noncoding RNA
SNAI2: snail family zinc finger 2
OSCC: oral squamous cell carcinoma
GISTs: gastrointestinal stromal tumors
HNF1A-AS1: lncRNA HNF1A-antisense 1
